# Adult psychiatric outcomes of young people who attended child and adolescent mental health services: a longitudinal total population study

**DOI:** 10.1017/S0033291724003568

**Published:** 2025-02-10

**Authors:** Colm Healy, Ulla Lång, Kirstie O’Hare, Johanna Metsälä, Karen O’Connor, Elaine Lockhart, Nicola Byrne, Juha Veijola, Eero Kajantie, Hugh Ramsay, Ian Kelleher

**Affiliations:** 1 Centre for Clinical Brain Sciences, Division of Psychiatry, University of Edinburgh, Edinburgh, UK; 2 School of Medicine, University College Dublin, Ireland; 3 Faculty of Medicine, University of Oulu, Oulu, Finland; 4 Population Health Unit, Finnish Institute for Health and Welfare, Helsinki, Finland; 5 RISE, Early Intervention in Psychosis Service & Home Based Treatment Team, South Lee Mental Health Services, Cork, Ireland; 6 Department of Psychiatry and Neurobehavioural Science, University College Cork, Cork, Ireland; 7 Royal Hospital for Children, Glasgow, Scotland, UK; 8 School of Health and Wellbeing, University of Glasgow, Scotland, UK; 9 Shine, Maynooth Business Campus, Kildare, Ireland; 10 Clinical Medicine Research Unit, MRC Oulu, University of Oulu and Oulu University Hospital, Oulu, Finland; 11 Department of Clinical and Molecular Medicine, Norwegian University of Science and Technology, Trondheim, Norway; 12 St John of God Hospitaller Services Group, Hospitaller House, Stillorgan, Dublin, Ireland

**Keywords:** childhood and adolescence, mental disorder, prediction and prevention, psychiatric services

## Abstract

**Background:**

There is an unprecedented societal focus on young people’s mental health, including efforts to expand access to child and adolescent mental health services (CAMHS). There has, however, been a lack of research to date to investigate adult mental health outcomes of young people who attend CAMHS.

**Methods:**

We linked Finland’s healthcare registries for all individuals born between 1987 and 1992. We investigated mental disorder diagnoses recorded in specialist adult mental health services (AMHS) and both inpatient and outpatient service use by age 29 (December 31, 2016) for former CAMHS patients.

**Results:**

Before the end of their 20s, more than half (52.4%, *n* = 21,183) of all CAMHS patients had gone on to attend AMHS. The most prevalent recorded adult psychiatric diagnoses received by former CAMHS patients were depressive disorders (30%, *n* = 11,768), non-phobic anxiety disorders (21%, *n* = 7,910), alcohol use disorders (9.5%, *n* = 3,427), personality disorders (9.3%, *n* = 3,366), and schizophrenia-spectrum disorders (7.6%, *n* = 2,945). In the total population, more than half of all AMHS appointments (53.1%, *k* = 714,239/1,345,060) were for former CAMHS patients. More than half of all inpatient psychiatry bed days were for former CAMHS patients (53.1%, *k* = 1,192,991/2,245,247).

**Conclusion:**

While there is a strong focus on intervening in childhood and adolescence to reduce the burden of mental illness, these findings suggest that young people who receive childhood intervention very frequently continue to require specialist psychiatric interventions in adulthood, including taking up a majority of both outpatient and inpatient service use. These findings highlight the need for a greater focus on research to alter the long-term trajectories of CAMHS patients.

## Introduction

The prevalence of mental health disorders in children and adolescents has been rising over the past three decades (Newlove-Delgado et al., 2022; Piao et al., 2022; Pitchforth et al., 2019; Malla et al., 2018). This has frequently been referred to as a youth mental health crisis and is recognized as one of the great challenges of our time (Viner et al., 2017; and McGorry et al., 2024). Furthermore, research demonstrating that mental health disorders frequently have their onset before age 18 (Solmi et al., 2022) has further highlighted the need for investment in mental health services for children and adolescents.

Child and adolescent mental health services (CAMHS) are well-established across most high-income countries (Signorini et al., 2018) and are an increasing priority for low- and middle-income countries (Simelane and de Vries, 2021). These services assess, diagnose, and treat mental health disorders of childhood, typically up to age 18 (Gerritsen et al., 2022; Anderson, Newlove-Delgado, & Ford, 2022). The long-term outcomes for patients of CAMHS, however, are unclear. Studies to date have followed only small numbers of individuals attending CAMHS and for relatively short periods (up to 2 years).

The Milestone study of transitions from CAMHS to adult mental health services (Gerristen et al., 2022) found that approximately 20% of patients who reached the upper age limit of CAMHS (16–18 years, depending on the specific service) went on to use adult mental health services. These figures, however, were based on just 763 young people followed up for just two years. Overall, there has been a lack of research to systematically evaluate adult psychiatric outcomes of CAMHS patients. This is essential information for understanding mental illness trajectories and real-world outcomes for children and adolescents who attend mental health services.

We used total Finnish population registry data to identify all individuals born 1987 to 1992 who, at some stage up to age 18 (2005 to 2010), attended CAMHS. We followed this CAMHS cohort up to age 29 to identify psychiatric service use outcomes in adulthood and mental disorder diagnoses received in adult mental health services.

## Method

### National Registry Data

Finnish national registry data were used to identify the population of interest. We linked data from the Medical Birth Registry (Birth Records), the Care Registry for Health Care (medical diagnosis and service setting), Statistics Finland (death record), and Digital and Population Data Services (emigration record). The Care Registry for Health Care provides information on all inpatient visits within a person’s lifetime and all outpatient visits to a secondary level of health care from the year 1998 to the present. Information on diagnosis (ICD-9 until 1995 and ICD-10 1996 to present), admission and discharge dates, and whether it was an inpatient or outpatient stay are recorded for all observations. The registry has been shown to have good diagnostic validity (Pihlajamaa et al., 2008; Sund, 2012). Data is reported in line with the STROBE guidelines (Von Elm et al., 2007).

### Population

All individuals born in Finland between 1987 and 1992 (*n* = 384,551) were included using the Medical Birth Registry. Only individuals who had died or emigrated prior to 2016 were excluded from the analysis (*n* = 11,064 2.9%), thus the final sample included all individuals born in Finland between 1987 and 1992 who had not died or emigrated prior to 2016, when the individuals were aged 25–30 (*n* = 373,487).

### Demographic variables

We report the sex observed at birth, and mother’s and father’s highest attained education at the time the index child was born. Low corresponds to International Standard Classification of Education (ISCED) classes 0 to 2, intermediate ISCED classes 3 to 5, and high ISCED classes 6 to 8 (UNESCO, 2011). Missing parental education data was reported as its own separate category.

### Exposure

#### CAMHS users

Within Finland, health care is available to all residents through a tax-funded system. The Care registry for health care records information on all specialist health care visit including the medical specialty of the treating doctor (i.e. psychiatry). This registry was used to identify all individuals who had attended child and adolescent mental health services. In addition, the format of the health care visit is also recorded (inpatient or outpatient) and this was used to identify all individuals who had an inpatient CAMHS admission prior to age 18.

### Outcomes

#### Mental disorders

Adult mental disorder diagnoses were identified using the Care Registry for Health Care. We extracted ICD section F one cipher (FX) and two ciphers (FXX) on all psychiatric diagnoses assigned in adulthood for ICD sections F0X-F9X.

#### Psychiatric service use

Adult inpatient and outpatient service use settings were identified using the care registry for health care. These were documented for all secondary or tertiary health care visits. Additionally, for inpatient admissions the duration of treatment was calculated as: discharge date − admission date + one day).

## Statistical analysis and procedure

### Adult mental disorder diagnosis

We report the incidence of any mental disorder diagnosed in adulthood and each category of mental disorder diagnosed in adulthood. We report CAMHS users’ cumulative risk and sensitivity for each of these outcomes. The cumulative risk was based on the net probability of each diagnosis and calculated by the end of follow up using *sts list* command in *Stata.* Using Cox regression, we report the hazard ratio for the association between CAMHS use with any diagnosis and each category of diagnosis in adulthood.

### Adult psychiatric service use

We report the number of adult outpatient appointments attended and adult inpatient admissions by CAMHS users and CAMHS non-users. For CAMHS users, we report the cumulative risk (net probability of going on to use adult service) and the sensitivity (total proportion of adult service use attributable to CAMHS users). Using Cox regression, we report the hazard ratio for the association between CAMHS use and (1) adult inpatient admission and (2) outpatient service use. We examine the proportion of multimorbidity across the diagnostic categories within those using adult services when stratified by CAMHS use. We compare the total number of days spent in adult inpatient treatment, and the median number of days per admission, between CAMHS users and CAMHS non-users.

### Supplementary results

Several additional analyses and sensitivity analyses were conducted. First, we report the incidence of multimorbidity across the diagnostic categories. Negative binomial regression was used to estimate the incidence rate ratio for the number of mental disorder categories observed for CAMHS users and CAMHS non-users. Second, we provide a further narrative report on the cumulative risk and sensitivity for (1) each category of mental disorder at the one-cipher level (FX), and (2) individual disorders at the two-cipher level (FXX) for individuals who had attended CAMHS. Third, we report the results for adult inpatient and outpatient service use for CAMHS users who had an inpatient admission before age 18. Fourth, we examined adult inpatient and outpatient service use for CAMHS users when stratified by sex. Fifth, we report the proportion of adult inpatient and outpatient service use attributable to each adult disorder category when stratified by CAMHS use. Finally, sixth, we report the adult inpatient and outpatient service use in childhood-onset (aged below 13) CAMHS users and adolescent onset (aged 13 and over) CAMHS users.

## Results

### Descriptive statistics

Of 373,487 individuals in the total population, 11.9% (*n* = 44,308) attended CAMHS at least once before age 18. The demographic characteristics of the CAMHS users and CAMHS non-users are reported in Table 1. Relative to CAMHS non-users, a greater proportion of CAMHS users were female (*X*^2^ = 1.8e^3^ *p* = 0.001). Low education rates were higher in mothers (*X*^2^ = 2.3e^3^ *p* < 0.001) and fathers (*X*^2^ = 1.2e^3^ *p* < 0.001) of CAMHS users compared to CAMHS non-users.

**Table 1.** Descriptive information for CAMHS users and CAMHS non-users

	CAMHS non-users % (*n*)	CAMHS users % (*n*)
**Overall *N* %**	88.1 (329,179)	11.9 (44,308)
**Male sex**	52.5 (172,688)	41.9 (18,556)
**Mothers highest education by birth**		
Low	18.8 (61,942)	28.1 (12,458)
Intermediate	68.5 (225,594)	62.6 (27,748)
High	11.5 (37,901)	8.4 (3,740)
Missing	1.1 (3,742)	0.8 (362)
**Fathers highest education by birth**		
Low	23.0 (75,548)	29.5 (13,054)
Intermediate	60.4 (198,927)	57.1 (25,316)
High	14.5 (47,793)	10.8 (4,775)
Missing	2.1 (6,911)	2.6 (1,163)

### Adult mental disorder diagnosis

#### Any diagnosis

By the end of follow up, 17.8% (*n* = 56,161) of the total population had received a mental health disorder diagnosis in specialist adult mental health services. For the cumulative risk by the end of follow up, sensitivity and hazard ratio of each mental disorder category see Table 2. In total, 52.4% of CAMHS users had at least one adult diagnosis by their late 20s (HR:6.5, CI:6.4–6.6). More than one-third (37.8%) of those with an adult diagnosis were former CAMHS patients.

**Table 2.** The adult incidence of each two cipher ICD 10 section F diagnosis (FXX) as well as the cumulative risk, sensitivity, and hazard ratio for disorder in those who have attended CAMHS relative to those who have not

ICD code	Diagnosis title	Adult population incidence %	Cumulative risk in CAMHs users %	Proportion of all diagnosis attributable to CAMHs %	Hazard ratio (95%ile CI)
*Fany*	*Any diagnosis*	*17.7*	52.4	37.8	6.5 (6.4–6.6)
*F0X*	*Organic, including symptomatic, mental disorders*	0.1	0.5	42.3	5.6 (4.5–7.0)
F00	Dementia in Alzheimer’s disease	<0.01^a^	<0.01^a^	66.7	14.9 (1.3–163.9)
F01	Vascular dementia	<0.01^a^	<0.01^a^	37.5	4.5 (1.1–19.0)
F02	Dementia in other diseases classified elsewhere	<0.1^a^	0.1	48.3	7.1 (4.3–11.8)
F03	Unspecified dementia	<0.01^a^	<0.01^a^	50.0	7.5 (1.5–37.0)
F04	Organic amnesic syndrome, not induced by alcohol and other psychoactive substances	<0.01^a^	<0.01^a^	>90.0^b^	N/A
F05	Delirium, not induced by alcohol and other psychoactive substances	<0.1^a^	0.3	40.9	5.3 (4.1–6.9)
F09	Unspecified organic or symptomatic mental disorder	<0.01^a^	<0.01^a^	35.3	4.1 (1.5–11.0)
*F1X*	*Mental and behavioral disorders due to psychoactive substance use*	*4.3*	*13.1*	37.7	4.8 (4.7–5.0)
F10	Alcohol	3.1	9.5	36.9	4.6 (4.4–4.8)
F11	Opioids	0.5	2.2	50.7	7.9 (7.2–8.8)
F12	Cannabinoids	0.6	2.0	41.2	5.4 (4.9–5.9)
F13	Sedatives or hypnotics	0.5	1.9	50.1	7.7 (6.9–8.6)
F14	Cocaine	<0.01^a^	<0.01^a^	35.3	4.1 (2.0–8.4)
F15	Other stimulants, including caffeine	0.4	1.5	50.0	7.7 (6.8–8.6)
F16	Hallucinogens	0.1	0.3	38.4	4.7 (3.7–6.1)
F17	Tobacco	0.1	0.5	35.5	4.3 (3.4–5.3)
F18	Volatile solvents	<0.01^a^	0.1	54.2	9.0 (5.1–15.9)
F19	Multiple drug use and use of other psychoactive substances	1.0	3.9	47.0	6.8 (6.4–7.3)
*F2X*	*Schizophrenia, schizotypal and delusional disorders*	*2.1*	*7.6*	*45.4*	*6.5 (6.2–6.9)*
F20	Schizophrenia	0.6	2.4	49.9	7.6 (7.0–8.4)
F21	Schizotypal disorder	0.1	0.6	51.5	8.0 (6.7–9.6)
F22	Persistent delusional disorder	0.1	0.5	48.2	7.1 (5.8–8.7)
F23	Acute and transient psychotic disorder	0.5	1.5	36.0	4.3 (3.9–4.8)
F24	Induced delusional disorder	<0.01^a^	<0.01^a^	50.0	7.6 (1.9–30.6)
F25	Schizoaffective disorder	0.2	0.9	52.8	8.5 (7.3–9.9)
F28	Other non-organic psychosis	0.1	0.4	56.2	9.8 (7.7–12.5)
F29	Unspecified non-organic psychosis	1.6	5.7	43.9	6.1 (5.8–6.5)
*F3X*	*Mood disorder*	*9.7*	32.4	42.0	6.6 (6.4–6.7)
F30	Manic episode	0.1	0.4	41.1	5.3 (4.2–6.6)
F31	Bipolar affective disorder	1.2	4.7	47.5	7.0 (6.6–7.5)
F32	Depressive episode	7.8	26.1	42.0	6.3 (6.2–6.5)
F33	Recurrent depressive episode	3.3	13.3	48.8	7.7 (7.4–8.0)
F34	Persistent mood disorder	0.8	3.4	51.6	8.2 (7.6–8.9)
F38	Other affective disorder	0.1	0.3	54.4	9.0 (6.8–11.9)
F39	Unspecified mood disorder	0.7	2.3	41.5	5.5 (5.0–6.0)
*F4X*	*Neurotic, stress-related, and somatoform disorders*	*9.4*	30.4	40.9	6.2 (6.0–6.3)
F40	Phobic disorders	1.7	6.6	46.8	6.9 (6.5–7.3)
F41	Other anxiety disorder	6.2	21.0	42.6	6.2 (6.0–6.4)
F42	Obsessive-compulsive disorder	0.7	2.9	49.6	7.6 (7.0–8.2)
F43	Reaction to severe stress, and adjustment disorders	2.9	9.7	41.4	5.6 (5.3–5.8)
F44	Dissociative disorder	0.2	1.1	56.3	9.8 (8.5–11.4)
F45	Somatoform disorder	0.4	1.3	33.1	3.8 (3.4–4.3)
F48	Other neurotic disorder	<0.1^a^	0.2	49.1	7.3 (5.4–9.8)
*F5X*	*Behavioral syndromes associated with physiological disturbances and physical factors*	*1.9*	7.4	47.9	7.2 (6.8–7.6)
F50	Eating disorders	1.1	5.0	54.7	9.3 (8.7–9.9)
F51	Non-organic sleep disorder	0.7	2.3	38.8	4.9 (4.5–5.4)
F52	Sexual dysfunction not of organic origin	<0.1^a^	0.1	21.4	2.1 (1.4–3.1)
F53	Mental and behavioral disorders associated with the puerperium	<0.1^a^	0.1	31.8	3.6 (2.6–5.0)
F54	Psychological and behavioral factors associated with disorders or diseases classified elsewhere	<0.1^a^	0.1	42.9	5.7 (3.7–9.0)
F55	Abuse of non-dependence-producing substances	<0.01^a^	<0.01^a^	71.4	18.9 (5.9–60.4)
F59	Unspecified behavioral syndromes associated with physiological disturbances and physical factors	<0.01^a^	<0.01^a^	57.1	10.0 (3.5–28.9)
*F6X*	*Disorders of adult personality and behavior*	2.3	10.0	52.0	8.6 (8.2–9.0)
F60	Specific personality disorder	1.9	8.3	54.1	9.3 (8.8–9.8)
F61	Mixed and other personality disorders	0.5	2.0	53.7	8.9 (8.0–9.9)
F62	Enduring personality changes, not attributable to brain damage and disease	<0.01^a^	<0.01^a^	63.0	12.9 (5.9–28.3)
F63	Habit and impulse disorders	0.1	0.4	46.4	6.6 (5.3–8.2)
F64	Gender identity disorders	0.2	0.6	36.3	4.3 (3.6–5.1)
F65	Disorders of sexual preference	<0.01^a^	0.0	57.1	10.0 (2.2–44.5)
F66	Psychological and behavioral disorders associated with sexual development and orientation	<0.01^a^	<0.01^a^	80.0	29.9 (11.2–79.6)
F68	Other disorders of adult personality and behavior	<0.01^a^	<0.01^a^	60.0	11.5 (5.2–25.6)
F69	Unspecified disorder of adult personality and behavior	<0.1^a^	0.1	50.0	7.6 (5.0–11.5)
F7X	Intellectual disability	0.4	1.0	30.2	3.3 (2.9–3.7)
F70	Mild intellectual disability	0.2	0.7	47.2	6.8 (5.7–8.0)
F71	Moderate intellectual disability	0.1	0.2	19.4	1.8 (1.4–2.4)
F72	Severe intellectual disability	<0.1^a^	<0.01^a^	9.5	0.8 (0.5–1.3)
F73	Profound intellectual disability	<0.1^a^	<0.01^a^	12.0	1.0 (0.5–2.0)
F78	Other intellectual disability	<0.1^a^	<0.01^a^	32.1	3.6 (1.6–7.9)
F79	Unspecified intellectual disability	0.1	0.1	22.2	2.2 (1.6–2.9)
*F8X*	*Disorders of psychological development*	*0.9*	*3.6*	*48.7*	*7.3 (6.8–7.8)*
F80	Specific developmental disorders of speech and language	0.1	0.4	39.5	4.9 (4.0–6.0)
F81	Specific developmental disorders of scholastic skills	0.3	1.1	47.6	6.9 (6.0–7.9)
F82	Specific developmental disorder of motor function	<0.1^a^	<0.1^a^	41.7	5.4 (3.0–9.5)
F83	Mixed-specific developmental disorders	0.1	0.4	50.8	7.8 (6.3–9.5)
F84	Pervasive developmental disorders	0.4	1.9	51.7	8.1 (7.3–9.0)
F88	Other disorders of psychological development	<0.1^a^	<0.01^a^	63.3	13.0 (6.2–27.3)
F89	Unspecified disorder of psychological development	<0.1^a^	0.1	44.3	6.0 (3.6–9.9)
*F9X*	*Behavioral and emotional disorders with onset usually occurring in childhood and adolescence*	*2.2*	*10.6*	*60.1*	*12.0 (11.4–12.6)*
F90	Hyperkinetic disorders	0.9	3.9	53.1	8.8 (8.1–9.5)
F91	Conduct disorders	0.1	0.6	84.4	40.4 (29.6–55.2)
F92	Mixed disorders of conduct and emotions	0.1	1.1	89.5	63.7 (48.3–84.0)
F93	Emotional disorders with onset specific to childhood	0.4	2.9	87.3	51.8 (44.4–60.5)
F94	Disorders of social functioning with onset specific to childhood and adolescence	<0.1^a^	0.2	81.9	33.8 (21.5–53.1)
F95	Tic disorders	0.1	0.4	59.1	10.9 (8.5–14.1)
F98	Other behavioral and emotional disorders with onset usually occurring in childhood and adolescence	0.2	1.0	54.9	9.3 (8.0–10.7)
*F99*	*Unspecified mental disorder*	*0.7*	*2.2*	*40.9*	*5.3 (4.9–5.8)*

*Note.*

a : Bottom coded to avoid statistical disclosure.

b : Top coded or not calculated to avoid secondary statistical disclosure.

#### Mental disorder categories

##### Cumulative risk

The cumulative risk of each mental disorder category ranged from 0.5% to 32.4%. The most common adult diagnoses in CAMHS users were depressive episode (*F*32, 26.1%), non-phobic anxiety disorder (*F*41, 21.0%), recurrent depressive episode (*F*33, 13.3%) severe stress, and adjustment disorder (*F*43, 9.7%) and alcohol use disorder (*F*10, 9.5%). Relative to the CAMHS non-users, there was an elevated risk of adult diagnosis across all mental disorder categories (HR range: 3.3–12.0).

##### Sensitivity

CAMHS users accounted for 30.2–60.1% of all mental disorder categories in the population. In terms of severe mental disorder, CAMHS users accounted for 45.4% of schizophrenia spectrum disorders (*F*2), 47.5% of bipolar affective disorder (*F*31), 54.1% of personality disorders (*F*60), and 48.8% of recurrent depressive disorders (*F*33).

### Outpatient adult mental health service use

#### Number and total proportion of all outpatient appointments attended

By age 29, 17.4% (*n* = 54,721) of the population had attended at least one adult mental health service outpatient appointment. The total number of outpatient appointments attended was *k* = 1,345,060. More than half (53.1% *k* = 714,239) of all outpatient adult mental health service appointments were by previous CAMHS users. CAMHS users had a higher number of adult outpatient appointments (see Supplementary Table 1). Compared to adult mental health service patients who had not previously attended CAMHS, CAMHS patients attended more than twice as many appointments (CAMHS users median:10 IQR:3–36; CAMHS non-users median:5 IQR:1–17; *X*^2^ = 318.8, *p* < 0.001). 59.9% (*n* = 12,479) of CAMHS users who attend adult outpatient services had adult disorders in multiple diagnostic categories, as compared to 42.5% (*n* = 14,414) adult outpatients without a history of CAMHS use (CAMHS users median disorder categories: 2 IQR:1–3; CAMHS non-users median disorder categories:1 IQR:1–2; *X*^2^ = 1.6e^3^, *p* < 0.001).

#### Cumulative risk and sensitivity

By the end of follow-up 51.6% of CAMHS users (n = 20,834) went on to use adult mental health service outpatient services, compared to 12.8% of CAMHS non-users (n = 33,887, HR:6.5 CI:6.4–6.6). 38.1% of all individuals who used adult outpatient services were CAMHS users.

### Inpatient adult psychiatry service use

#### Number and total proportion of all inpatient admissions

By age 29, 5.8% of the cohort (*n* = 14,474) had one or more adult psychiatric inpatient admission. The total number of inpatient admissions for the population was *k* = 45,471. In total, 52.2% (*k* = 23,728) of all adult inpatient admissions were by previous CAMHS patients.

CAMHS users had a higher number of adult inpatient admissions compared to CAMHS non-users (see Table 3). Limiting the analyses to individuals who had one or more adult inpatient admissions, former CAMHS users had a median of two adult inpatient admissions, compared to one for those who had not previously attended CAMHS (CAMHS Median:2 IQR:1–4; and CAMHS non-users Median:1 IQR:1–3; *X*^2^ = 224.6, *p* < 0.001). 83.7% (*n* = 5,278) of CAMHS users who used adult inpatient services had adult disorders in multiple diagnostic categories, as compared to 67.7% (*n* = 5,531) of adult inpatients without a history of CAMHS use (CAMHS users median disorder categories: 3 IQR:2–4; CAMHS non-users median disorder categories:2 IQR:1–3; *X*^2^ = 566.7, *p* < 0.001).

**Table 3.** Percentage and odds ratio for the number adult inpatient admission in CAMHS users relative to CAMHS non-users

Adult psychiatric service use	CAMHS non-users % (*n*)	CAMHS users % (*n*)	Odds ratio (95%CI)
0	97.5%	85.8%	Ref
	(321,012)	(38,001)	
1	1.3%	5.6%	5.**1**
	(4,125)	(2,501)	(4.9–5.4)
2–5	1.0%	6.1%	7.**0**
	(3,269)	(2,711)	(6.7–7.4)
6 +	0.2%	2.5%	12.**0**
	(773)	(1,095)	(10.9–13.1)

#### Cumulative risk and sensitivity

22.5% of CAMHS users (*n* = 6,307) went on to have an adult inpatient admission compared to 3.8% of CAMHS non-users (*n* = 8,167, HR:6.3 CI:6.1–6.6). In total, 43.6% of all individuals who had an adult inpatient admission were former CAMHS users.

#### Duration of admission

CAMHS users accounted for 53.1% (*k* = 1,192,991) of the 2,245,247 adult psychiatric inpatient bed days observed in this cohort. Among individuals who had one or more adult psychiatry inpatient admission, former CAMHS users had a higher number of inpatient treatment days in adulthood (CAMHS users median: 72 days IQR:9–233; CAMHS non-users median: 48 days IQR:5–163; *X*^2^ = 88.4, *p* < 0.001) as well as a higher number of days per admission (CAMHS users median: 36 days IQR:7–84; CAMHS non-users median: 32 days IQR:5–81; *X*^2^ = 40.1, *p* < 0.001).

### Supplementary analysis

Full information on all Supplementary Analyses is in Supplementary Materials. Below we briefly highlight some important observations.

#### Multimorbidity across diagnostic categories

28.5% of CAMHS users had diagnoses from multiple diagnostic categories in adulthood. For information on the incidence of multimorbidity across the diagnostic categories see Supplementary Analysis 1.

#### CAMHS inpatient users and adult mental health service use

In total, 2.8% (*n* = 10,303) of the population had one or more inpatient CAMHS admission. This group accounted for almost 30% (29.2%) of all adult inpatient psychiatry admissions, 30.8% of all adult inpatient bed days, and 22.7% of adult outpatient mental health service appointments. For full information on the adult mental health service use of CAMHS users who had one or more inpatient admissions before age 18, see Supplementary Analysis 3 and Supplementary Tables 2 and 3.

#### CAMHS users adult mental health service use when stratified by sex

Female CAMHS users accounted for 59.8% of all female adult inpatient admissions and outpatient appointments (57.5%). For full information on CAMHS users adult mental health service use when stratified by sex, see Supplementary Analysis 4, Supplementary Tables 4 and 5.

#### Adult mental health service use in child (under 13) and adolescent (13 and over) CAMHS users

Adolescent CAMHS users accounted for 39.6% of all adult inpatient admissions. For full results see Supplementary Analysis 6, Supplementary Tables 7 and 8.

For extended reporting on the diagnoses in each mental disorder category, see Supplementary Analysis 2. For results on the proportion of adult psychiatry service use attributable to each disorder when stratified by CAMHS use, see Supplementary Analysis 5 and Supplementary Table 6.

## Discussion

We have documented detailed recorded mental health diagnostic and service use outcomes for a total population of young people who attended child and adolescent mental health services followed into adulthood. By age 29 years, 52% of CAMHS patients had one or more appointments with specialist adult mental health services. The most prevalent diagnoses recorded in adult mental health services by patients who had previously attended CAMHS were depressive episodes (30%, including depressive episodes and recurrent depression), non-phobic anxiety disorders (21.0%, including generalized anxiety disorder, panic disorder, and mixed anxiety disorders), alcohol use disorders (9.5%), personality disorders (9.3%), and schizophrenia-spectrum disorders (7.6%).

There were marked differences in adult mental health service use by patients with a history of CAMHS attendance compared to adult mental health patients with no history of CAMHS attendance. Specifically, individuals with previous CAMHS attendance had double the total number of outpatient adult mental health appointments (median 5 vs 10). In fact, more than half of all outpatient appointments (53.1%) in specialist adult mental health services were taken up by patients who had previously attended CAMHS.

Former CAMHS use was also predictive of greater adult inpatient service use. In total, 22.5% of former CAMHS patients had one or more adult psychiatric inpatient admission. Compared to those with no history of CAMHS attendance, individuals who had previously attended CAMHS had a greater number of total days as an inpatient (median 48 vs 72 days). Overall, more than half (53.1%) of all inpatient days in specialist adult mental health services were taken up by patients who had previously attended CAMHS.

A key finding of the current study was that risk for many of the most severe mental illnesses of adult life was captured in CAMHS, many years before their onset.,). Looking at some of the most severe mental disorders across the lifespan, previous meta-analytic research has shown that just 13% of bipolar affective disorders, 12% of recurrent depressive disorders, 10% of personality disorders, and 8% of schizophrenia cases are diagnosed by age 18 (Solmi et al., 2022). In the current study, however, we found that these disorders were very frequently preceded by help-seeking in childhood for other mental health problems. Approximately half of the total population of recorded diagnoses of schizophrenia, recurrent depressive disorder, bipolar affective disorder, and personality disorders occurred in patients who had attended CAMHS. This highlights that, although these disorders may typically emerge in adulthood, they are frequently preceded by help-seeking for (heterotypic) pediatric mental health problems in real-world psychiatry services). These findings also highlight enormous opportunities for prediction and, ultimately, prevention of many of the most severe and enduring disorders of adult life, risks that are automatically captured within existing child and adolescent mental health services).

Our findings also highlight the need for specific research on childhood interventions to reduce the risk for later severe mental illnesses. Whilst evidence-based interventions are used in CAMHS to treat childhood mental health disorders, it is not clear what effect, if any, these treatments have on longer-term mental health trajectories and adult psychiatric outcomes (Roest et al., 2023). Recent findings from the Great Smoky Mountains cohort study, for example, showed that real-world child mental health services were not associated with a reduced risk of adult mental health disorders when followed up to age 30 years (Copeland et al., 2022). A specific evidence base is needed to understand the effect of childhood interventions on longer-term mental health trajectories and adult mental health outcomes, which is currently lacking. That is, in addition to research looking at the direct effects of mental health treatments administered for childhood mental disorders, which is the typical focus of intervention research, investment is also needed in a new area of mental health science, focused on how childhood interventions may impact on longer-term mental health outcomes, including the prevention of severe mental disorders of adulthood such as personality, mood, and psychotic disorders).

This study has several strengths, including that it captured all specialist public psychiatry services. Private psychiatric treatment is missing from the data, but private services make up only a small proportion of hospital and specialist care in Finland. Our study used health register data, thus, it included only individuals presenting to specialist mental health services and did not identify all psychopathology in the general population. This, however, is precisely the point of this approach: our aim was not to investigate childhood mental disorders as a risk factor for adult psychopathology or mental health service use. Rather, it was to assess how contact with child and adolescent mental health services captures risk for later recorded mental disorder and service use. Our study did not have counterfactual data that would allow us to investigate adult outcomes for young people with similar mental health problems who did not present to CAMHS. This, however, does not detract from our ability to identify adult psychiatric outcomes of former CAMHS patients.

## Conclusion

More than half of all adult psychiatric outpatient appointments and inpatient bed days from age 18 to 29 were taken up by individuals who had previously attended specialist child and adolescent mental health services, indicating the long-term clinical and functional impact predicted by mental health help-seeking in childhood. Attendance of CAMHS, while uncommonly associated with severe contemporaneous mental illness, is frequently associated with subsequent chronic and enduring mental disorders in adulthood, including capturing approximately half of all recorded cases of schizophrenia, bipolar disorder, recurrent depression, and personality disorders diagnosed up to age 29. This demonstrates that severe mental illness trajectories are commonly captured early in life in child and adolescent mental health services. It also demonstrates the enormous opportunities for the prediction and prevention of severe mental illness that naturally arise in existing specialist CAMHS. We currently lack evidence as to how to alter these long-term mental health trajectories. Our findings highlight the need for major investment in a new area of prevention mental health science focused on long-term outcomes of children and adolescents presenting with mental health problems.

## Supporting information

Healy et al. supplementary material Healy et al. supplementary material

## Supplementary material

The supplementary material for this article can be found at http://doi.org/10.1017/S0033291724003568.
