# Supplementary material for: Adult psychiatric outcomes of young people who attended child and adolescent mental health services: a longitudinal total population study
Source: Psychol Med. 2025 Feb 10;55:e34. doi: 10.1017/S0033291724003568 (PMC12017351; doi:10.1017/S0033291724003568)
Supplement: Healy et al. supplementary material [file S0033291724003568sup001.docx]

S**upplementary Materials.**

**Table of Content.**

| **Content** | **Title** | **Page** |
| --- | --- | --- |
| **Supplementary Analysis 1.** | Diagnostic category multimorbidity when stratified by CAMHS attendance. | **2** |
| **Supplementary Figure 1.** | Percentage of individuals with diagnosis from multiple diagnostic categories when stratified by CAMHS attendance. | **2** |
| **Supplementary Analysis 2.** | Cumulative risk and Sensitivity for the individual diagnosis with each category. | **3** |
| **Supplementary Table 1.** | Percentage and odds ratio for the number of adult outpatient appointments attended in CAMHS users relative to CAMHS non-users | **5** |
| **Supplementary Analysis 3.** | CAMHS inpatient users and adult psychiatric service use. | **6** |
| **Supplementary Table 2.** | Percentage and odds ratio for adult inpatient admission in CAMHS non-users and CAMHS non-inpatient users relative to CAMHS inpatient users. | **6** |
| **Supplementary Table 3.** | Percentage and odds ratio for the number of adult outpatient appointments attended in CAMHS non-users and CAMHS non-inpatient users relative to CAMHS inpatient users. | **7** |
| **Supplementary Analysis 4.** | CAMHS users adult psychiatric service use when stratified by sex. | **8** |
| **Supplementary Table 4.** | Percentage and odds of adult inpatient admissions when stratified by sex and CAMHS usage. | **8** |
| **Supplementary Table 5.** | Percentage and odds of adult outpatient service use by category in those CAMHS and non-CAMHS users when stratified by male and female individuals. | **9** |
| **Supplementary Analysis 5.** | The proportion of adult psychiatric service use attributable to each disorder category and when stratified by CAMHS use. | **10** |
| **Supplementary Table 6.** | The proportion of adult psychiatric services attributable to each disorder category when stratified by CAMHS use | **10** |
| **Supplementary Analysis 6.** | Adult psychiatric service use in childhood onset (under 13) and adolescent onset (13 and older) CAMHS users. | **12** |
| **Supplementary Table 7.** | Percentage and odds ratio for adult inpatient admission in CAMHS non-users and CAMHS non-inpatient users relative to CAMHS inpatient users. | **12** |
| **Supplementary Table 8.** | Percentage and odds ratio for the number of adult outpatient appointments attended in CAMHS non-users and CAMHS non-inpatient users relative to CAMHS inpatient users. | **13** |

**Supplementary Analysis 1. *Diagnostic category multimorbidity when stratified by CAMHS attendance.***

**Supplementary Figure 1**. Percentage of individuals with diagnosis from multiple diagnostic categories when stratified by CAMHS attendance.

*Diagnostic Category Multimorbidity.* 19·5% of CAMHS users (n=8,640) went on to have an adult diagnosis from a single diagnostic category, 13·6% (n=6,023) had diagnoses from two diagnostic categories and 14·9% (n=6,587) from three or more diagnostic categories (see supplementary Figure 1). Whereas only 6·1% of CAMHS non-users (n=20,291) went on to have an adult diagnosis in a single diagnostic category and 2·7% (n=8,831) had diagnoses from two diagnostic categories and only 1·8% (n=5,789) had diagnoses from three or more diagnostic categories in adulthood. Attending CAMHS was associated with 5·8-fold greater risk of having multiple (two or more) diagnostic categories in adulthood (IRR; 5·8, CI:5·7-5·8).

***Supplementary analysis 2. Cumulative risk and Sensitivity for the individual diagnosis with each category.***

In almost all circumstance, relative to CAMHS non-users, CAMHS users had an elevated risk of adult mental disorders across all categories (HR range: 3·3-12·0).

*F0X Organic, including symptomatic, mental disorders.* The adult incidence of each organic mental disorders by age 29 was low (all F0X <0·1). Less than 0·5% of CAMHS users had a F0X diagnoses. Most common was Delirium, not induced by alcohol or other psychoactive substances. CAMHS users accounted for a large proportion of all organic disorder cases (range 35·3%- >90%).

*F1X Mental and behavioural disorders due to psychoactive substance use.*  9·5% of CAMHS users go on to have an adult alcohol related disorders and 3·9% a poly-substance use disorder. Less than 2·5% of CAMHS users go on to have an adult disorder associated with other substances. CAMHS users accounted for over a third all substance use diagnosis cases and this rises to 54% for cases involving volatile solvents.

*F2X Schizophrenia, schizotypal and delusional disorders.* 5·7% of CAMHS users go on to have an adult diagnosis of unspecified non-organic psychosis while 2·4% are diagnosed with schizophrenia. Very few CAMHS attendees had other F2X diagnoses (range:<0·01- 1·5%). CAMHS users account for a large proportion of all adult psychosis cases, ranging from 36% of acute and transient psychotic disorders cases to 56·2 of other non-organic psychosis cases.

*F3X Mood disorders.* 26·1% of CAMHS users have a depressive episode in adulthood, 13·3% have recurrent depressive episodes and 4·7% have a diagnosis of bi-polar disorder. CAMHS users account for a large proportion of all mood disorder diagnosis cases (range: 41·1% - 54·4%).

*F4X Neurotic, stress-related and somatoform disorders*. 21·0% of CAMHS users go on to have a diagnosis of other anxiety disorder in adulthood. 6·6% have an adult diagnosis of a phobic disorder, and 2·9% have an adult diagnosis of obsessive-compulsive disorder. With the except for somato-form disorders (33·1%), CAMHS users account for a large proportion of all disorder categories (42·6-56·3%).

*F5X Behavioral syndromes associated with physiological disturbances and physical factors.* Some disorders with this category had very low incidence. 5·0% of CAMHS users had an adult diagnosis of eating disorder, and 2·3% had a diagnosis of non-organic sleep disorder. CAMHS users account for over 50% for unspecified behavioral syndrome cases, eating disorder cases, abuse of non-dependence-producing substances cases but only 21·4% of sexual dysfunction not of organic origin case and 31·8% for mental disorder associated with the puerperium case. Very few CAMHS user (<0·1%) had other F5X disorders in adulthood.

*F6X Disorders of adult personality and behavior.* 8·3% of CAMHS users had an adult specific personality disorder but very few CAMHS users reported all other diagnosis within this category (<2·0 CAMHS users account for a high proportion of all cases within this category (36·3-80·0%).

*F7X Intellectual Disability.* The adult incidence of intellectual disability was very low (0·4%). 1% of CAMHS users had adult diagnosis of intellectual disability. CAMHS users accounted for between 9·5% and 47·2% of all cases across each diagnoses. The lowest was for profound intellectual disability the highest was for mild intellectual disability.

*F8X Disorders of Psychological Development.* The adult incidence of disorders of psychological development was very low (<0·4%). 1·7% of CAMHS users had a pervasive developmental disorder. CAMHS users accounted for a large proportion of all diagnoses within this category (range: 39·5%-63·3%)

F9X Behavioural and emotional disorders with onset usually occurring in childhood and adolescence. 2·2% of the population had an F9 category diagnosis. 3·9% of CAMHS users had an adult incidence of hyperkinetic disorder and 2·9% had emotional disorder. CAMHS users accounted for a very high proportion of all cases across this diagnostic category (53·1%-89·5%).

F99. Unspecified mental disorder. 2·2% of CAMHS user had an adult incidence of unspecified mental disorder. CAMHS users accounted for 40·9% of adult incidence of unspecified mental disorders.

**Supplementary Table 1.** Percentage and relative risk ratio for the number of adult outpatient appointments attended in CAMHS users relative to CAMHS non-users

| **Number of adult outpatient appointments attended.** | **CAMHS non-users % (n)** | **CAMHS users**  **% (n)** | **Risk Ratio**  **(95%CI)** |
| --- | --- | --- | --- |
| **0** | 89·7  (295,292) | 53·0  (23,474) | Ref |
| **1** | 2·9  (9,498) | 7·3  (3,219) | **4**·**3**  (4·1-4·4) |
| **2-5** | 2·7  (8,758) | 10·1  (4,455) | **6**·**4**  (6·2-6·6) |
| **6-10** | 1·2  (4,066) | 6·2  (2,751) | **8**·**5**  (8·1-8·9) |
| **11-25** | 1·6  (5,174) | 8·5  (3,785) | **9**·**2**  (8·8-9·6) |
| **26-50** | 1·0  (3,139) | 6·0  (2,647) | **10**·**6**  (10·1-11·2) |
| **51-100** | 0·6  (1,997) | 4·8  (2,128) | **13**·**4**  (12·6-14·3) |
| **101+** | 0·4  (1,255) | 4·2  (1,849) | **18**·**5**  (17·2-19·9) |

**Supplementary analysis 3. CAMHS inpatient users and adult psychiatric service use.**

Of those who attended CAMHS, 23·3% (n=10,303) required an inpatient admission prior to their 18^th^ birthday.

**Adult Inpatient services.** *Number and total proportion of all inpatient admission.* The total number of adult inpatient admissions was k=45,471. 29·2% (k=13,266) of these adult admissions were from previous CAMHS users who required an inpatient admission before 18. A significantly higher percentage of CAMHS inpatient users had an adult inpatient admission relative to CAMHS non-users (HR:13·1, CI:12·6-13·7) and CAMHS users who did not have a childhood inpatient admission (HR:2·9, CI:2·8-3·1). CAMHS inpatient users also have a vastly higher number of adult inpatient admissions than non-CAMHS users and CAMHS users who did not have a childhood inpatient admission (See table 2).

**Supplementary Table 2.** Percentage and relative risk ratio for adult inpatient admission in CAMHS non-users and CAMHS non-inpatient users relative to CAMHS inpatient users.

| **Number of adult Inpatient admissions** | **CAMHS non-user**  **% (n)** | **CAMHS non-inpatient Users % (n)** | **CAMHS -inpatient Users % (n)** | **CAMHS inpatient user comparison** | |
| --- | --- | --- | --- | --- | --- |
|  |  |  |  | **CAMHS non-Users**  **Risk Ratio**  **(95%CI)** | **CAMHS non-inpatient Users Risk Ratio**  **(95%CI)** |
| 0 | 97·5  (321,012) | 89·7  (30,485) | 73·0  (7,516) | Ref | Ref |
| 1 | 1·3  (4,125) | 4·8  (1,629) | 8·5  (872) | **9**·**0**  (8·4-9·7) | **2**·**2**  (2·0-2·4) |
| 2-5 | 1·0  (3,269) | 4·3  (1,454) | 12·2  (1,257) | **16**·**4**  (15·3-17·6) | **3**·**5**  (3·2-3·8) |
| 6 + | 0·2  (773) | 1·3  (437) | 6·4  (658) | **36**·**4**  (32·6-40·4) | **6**·**1**  (5·4-6·9) |

*Duration of stay and age of first admission.* CAMHS inpatient users have a higher number of days spend in inpatient treatment in adulthood (CAMHS inpatient users median:104 days IQR:17-312; CAMHS non-inpatient users median:60 days IQR:7-184; CAMHS non-users median:60 days IQR:7-184; X^2^ = 172·9, p < 0·001) as well as a higher number of days per admission (CAMHS inpatient users median:43 days IQR:11-90; CAMHS non-inpatient users median:32 days IQR:5-80; CAMHS non-users median:32 days IQR:5-81; Chi2= 90·1, p <0·001). CAMHS inpatient users account for 30·8% of adult psychiatric inpatient bed days (k=691,750). CAMHS inpatient users have their first adult inpatient admission roughly one year earlier than CAMHS non-inpatient users and two years early than CAMHS non-users (CAMHS inpatient users median age:20·0 IQR:18·6-22·4; CAMHS non-inpatient users median age:21.2 IQR:19·4-23·5; and CAMHS non- users median age:22·4 IQR:20.2-24·6, X^2^ = 551·0, p < 0·001).

**Outpatient service use.**  *Number and total proportion of all outpatient appointments attended.* The total number of outpatient appointments attended was k=1,345,060. 22·7% (k= 305,903) of these appointments were from previous CAMHS users who required an inpatient admission before 18. A significantly higher percentage of CAMHS inpatient users used adult outpatient services relative to CAMHS non-users (HR:10·8, CI:10·5-11·1) and CAMHS users who did not have a childhood inpatient admission (HR:1·9, CI:1·9-2·0). CAMHS inpatient users also have a vastly higher number of adult outpatient appointments than non-CAMHS users and CAMHS users who did not have a childhood inpatient admission (See table 3).

**Supplementary Table 3.** Percentage and relative risk ratio for the number of adult outpatient appointments attended in CAMHS non-users and CAMHS non-inpatient users relative to CAMHS inpatient users.

| **Number of adult Inpatient admissions** | **CAMHS non-user**  **% (n)** | **CAMHS non-inpatient Users % (n)** | **CAMHS -inpatient Users % (n)** | **CAMHS inpatient user comparison** | |
| --- | --- | --- | --- | --- | --- |
|  |  |  |  | **CAMHS non-Users**  **Risk Ratio**  **(95%CI)** | **CAMHS non-inpatient Users Risk Ratio**  **(95%CI)** |
| **0** | 89·7  (295,292) | 57·8  (19,648) | 37·1  (3,826) | Ref | Ref |
| **1** | 2·9  (9,498) | 7·1  (2,427) | 7·7  (792) | **6**·**4**  (5·9-7·0) | **1**·**7**  (1·5-1·8) |
| **2-5** | 2·7  (8,758) | 9·4  (3,210) | 12·1  (1,245) | **11**·**0**  (10·3-11·7) | **2**·**0**  (1·8-2·1) |
| **6-10** | 1·2  (4,066) | 5·7  (1,976) | 7·5  (775) | **14**·**7**  (13·5-16·0) | **2**·**0**  (1·8-2·2) |
| **11-25** | 1·6  (5,174) | 7·9  (2,689) | 10·6  (1,096) | **16**·**3**  (15·2-17·8) | **2**·**1**  (1·9-2·3) |
| **26-50** | 1·0  (3,139) | 5·2  (1,781) | 8·4  (866) | **21**·**3**  (19·6-23·1) | **2**·**5**  (2·3-2·7) |
| **51-100** | 0·6  (1,997) | 3·9  (1,316) | 7·9  (812) | **31**·**4**  (28·8-34·3) | **3**·**2**  (2·9-3·5) |
| **101+** | 0·4  (1,255) | 2·8  (958) | 8·7  (891) | **54**·**8**  (50·0-60·1) | **4**·**8**  (4·3-5·3) |

*Age of first admission.* CAMHS inpatient users have their first adult outpatient appointment roughly 6 months year earlier than CAMHS non-inpatient users and over three years early than CAMHS non-users (CAMHS inpatient users median age:18·4 IQR:18·1-20·7; CAMHS non-inpatient users median age:18·9 IQR:18·1-21·8; and CAMHS non-users median age:22·2 IQR:20·1-24·5, X^2^ = 6700, p < 0·001).

***Supplementary analysis 4. CAMHS users adult psychiatric service use when stratified by sex.***

**Male Inpatient admissions.** Within males within this cohort by age 29, 3·6% individuals (n= 6,928) required an adult inpatient admission. The total number of admissions was k=20,342. 42·7% (k= 8683) of these admissions were from male CAMHS users. 13·7% of male CAMHS users go on to have an adult inpatient admission, relative to only 2·3% of CAMHS non-users (n= 4,386, OR:6·1 CI:5·8-6·4). Even after adjusting for inpatient admission before 18, male CAMHS users still have 4.5-time greater odds of an adult inpatient admission (OR:4·3 CI: 4·1-4·6). 36·7% (n= 2,542) of all males who have had an adult inpatient admission were CAMHS users.

**Female inpatient admissions.** Within females within cohort and by age 29, 4·1% individuals (n= 7,546) required an adult inpatient admission. The total number of admissions was k=25,129. Almost 60% (59·8% k= 15,045) of these observations were from female CAMHS users. 14·6% of female CAMHS users go on to have an adult inpatient stay, relative to only 2·1% of female CAMHS non--users (n= 3,781, OR:6·9 CI:6·6-7·2). Even after adjusting for inpatient admission before 18, female CAMHS users still have 4·5-time greater odds of an adult inpatient admission (OR:4·7 CI: 4·5-5·0). 49·9% (n=3,765) of all females who have had an adult inpatient admission were CAMHS users.

**Supplementary Table 4.** Percentage and odds of adult inpatient admissions when stratified by sex and CAMHS usage.

| **Number of adult Inpatient admissions** | **Males** | | | **Females** | | |
| --- | --- | --- | --- | --- | --- | --- |
|  | **Non CAMHS user**  **% (n)** | **CAMHS user**  **% (n)** | **Odds Ratio**  **(95%CI)** | **Non CAMHS user**  **% (n)** | **CAMHS user**  **% (n)** | **Odds Ratio**  **(95%CI)** |
| 0 | 97·5%  (168,302) | 86·3%  (16,014) | Ref | 97·6%  (152,686) | 85·4%  (21,987) | Ref |
| 1 | 1·3%  (2,171) | 5·5%  (1,022) | **4**·**9**  (4·6-5·3) | 1·3%  (1,954) | 5·7%  (1,479) | **5**·**3**  (4·9-5·6) |
| 2-5 | 1·0%  (1,789) | 6·0%  (1,116) | **6**·**6**  (6·1-7·1) | 1·0%  (1,480) | 6·2%  (1,595) | **7**·**5**  (7·0-8·0) |
| 6 + | 0·3%  (426) | 2·2%  (404) | **10**·**0**  (8·7-11·4) | 0·2%  (347) | 2·7%  (691) | **13**·**8**  (12·1-15·7) |

**Male Outpatient appointments attended.**  Within this cohort, 12·1% of males (n=23,169) attended adult outpatient appointments and accounted for 483,057 individual appointments. 45·3% of these appointments were from males who have been to CAMHS (n= 218,887). Male CAMHS users accounted for 32·2% (n= 7,465) of males who have had used adult outpatient services. 40·2% of males who went to CAMHS go on to use adult outpatient services, relative to only 9·1% (n= 15,704) of male non-CAMHS users (OR:6·7 CI:6·5-7·0).

**Female Outpatient appointments attended.**  Within this cohort, 17·3% of females (n=31,552) attended adult outpatient appointments and accounted for 862,003 individual appointments. Over half of these appointments are from females who have been to CAMHS (57·5% n= 495,352). Female CAMHS users accounted for 42·4% (n= 13,369) of females who have had used adult outpatient services. 51·9% of females who went to CAMHS go on to use adult outpatient services, relative to only 11·6.% (n= 18,183) of female non-CAMHS users (OR:8·2 CI:8·0-8·5).

**Supplementary Table 5.** Percentage of Adult Outpatient service use by category in those CAMHS and non-CAMHS users when stratified by male and female individuals.

| **Number of adult outpatient appointments attended.** | **Male** | | | **Female** | | | |
| --- | --- | --- | --- | --- | --- | --- | --- |
|  | **Non-CAMHS user % (n)** | **CAMHS user**  **% (n)** | **Odds Ratio**  **(95%CI)** | **Non-CAMHS user % (n)** | **CAMHS user**  **% (n)** | **Odds Ratio**  **(95%CI)** |  |
| **0** | 90·9  (156,984) | 59·8  (11,091) | Ref | 88·4  (138,284) | 48·1  (12,383) | Ref |  |
| **1** | 2·9  (5,069) | 8·0  (1,478) | **4**·**1**  (3·9-4·4) | 2·8  (4,429) | 6·8  (1,741) | **4**·**4**  (4·1-4·7) |  |
| **2-5** | 2·4  (4,131) | 9·3  (1,728) | **5**·**9**  (5·6-6·3) | 3·0  (4,627) | 10·6  (2,727) | **6**·**6**  (6·3-6·9) |  |
| **6-10** | 1·0  (1,821) | 5·5  (1,017) | **7**·**9**  (7·3-8·6) | 1·4  (2,245) | 6·7  (1,734) | **8**·**6**  (8·1-9·2) |  |
| **11-25** | 1·2  (2,075) | 6·8  (1,256) | **8**·**6**  (8·0-9·2) | 2·0  (3,099) | 9·8  (2,529) | **9**·**1**  (8·6-9·6) |  |
| **26-50** | 0·7  (1,264) | 4·4  (818) | **9**·**2**  (8·4-10·0) | 1·2  (1,875) | 7·1  (1,829) | **10**·**9**  (10·2-11·6) |  |
| **51-100** | 0·5  (815) | 3·3  (612) | **10**·**6**  (9·6-11·8) | 0·8  (1,182) | 5·9  (1,516) | **14**·**3**  (13·2-15·5) |  |
| **101+** | 0·3  (529) | 3·0  (556) | **14**·**9**  (13·1-16·8) | 0·5  (726) | 5·0  (1,293) | **19**·**9**  (18·1-21·8) |  |

***Supplementary analysis 5. The proportion of adult psychiatric service user attributable to each disorder category and when stratified by CAMHS use.***

Within this investigation we examined the percentage contribution of each disorder category to the total percentage of those who attend adult psychiatric services and when stratified by CAMHS users and CAMHS non-users. Percentages do not sum to 100 due to concurrent and sequential co-morbidity. Almost all who attend adult psychiatric service use have a psychiatric disorder in adulthood (overall: 94·3%, inpatient: 97·8 and outpatient: 93·1, see Supplementary Table 6). The most common mental disorders observed in adult psychiatric services (any, inpatient and outpatient) were mood disorders and neurotic disorders. The results indicated that 22·8% of adult psychiatric service use was attributable to those with a mood disorder with a history of CAMHS use. 31·5% of adult psychiatric service use was attributable to those with a mood disorder without a history of CAMHS use. Beyond mood and neurotic disorders, other proportional discrepancies between CAMHS users and non-users were >3%, however it should be noted that CAMHS users only account for <12% of the total population and CAMHS non-users account for >88% of the population. Similar results were observed when the sample was limited to inpatient service use and outpatient service use only.

**Supplementary Table 6.** The proportion of adult psychiatric services attributable to each disorder category and when stratified by CAMHS use

| Disorder Category | ICD-10 Code | Adult Psychiatric Service use % | CAMHS users %^a^ | | CAMHS non-users %^a^ |
| --- | --- | --- | --- | --- | --- |
| Any Psychiatric Service Use (n=55,951) |  |  |  | |  |
| Any Diagnosis in Adult Services |  | 99·9 | 37·8 | 62·1 | |
| Organic Disorders | F00-F09 | 0·6 | 0·2 | 0·3 | |
| Substance use Disorders | F10-F19 | 10·3 | 4·5 | 5·8 | |
| Schizophrenia Spectrum Disorder | F20-F29 | 11·6 | 5·3 | 6·3 | |
| Mood Disorders | F30-F39 | 54·3 | 22·8 | 31·5 | |
| Neurotic Disorders | F40-F48 | 51·1 | 20·9 | 30·1 | |
| Eating Disorder | F50-F59 | 10·8 | 5·2 | 5·6 | |
| Specific Personality Disorder | F60-F69 | 12·5 | 6·5 | 6·0 | |
| Intellectual Disabilities | F70-F79 | 2·1 | 0·6 | 1·5 | |
| Pervasive developmental disorders | F80-F89 | 5·1 | 2·5 | 2·6 | |
| Behavioral Disorders | F90-F98 | 9·4 | 6·3 | 3·1 | |
| Inpatient Service Use (n=14,474) |  |  |  |  | |
| Any Diagnosis |  | >99·9 | 43·5 | 56·5 | |
| Organic Disorders | F00-F09 | 1·6 | 0·7 | 0·9 | |
| Substance use Disorders | F10-F19 | 26·2 | 12·1 | 14·1 | |
| Schizophrenia Spectrum Disorder | F20-F29 | 33·1 | 15·0 | 18·1 | |
| Mood Disorders | F30-F39 | 64·3 | 31·1 | 33·1 | |
| Neurotic Disorders | F40-F48 | 52·2 | 26·2 | 26·0 | |
| Eating Disorder | F50-F59 | 12·8 | 7·0 | 5·8 | |
| Specific Personality Disorder | F60-F69 | 25·4 | 14·4 | 10·9 | |
| Intellectual Disabilities | F70-F79 | 3·3 | 1·2 | 2·1 | |
| Pervasive developmental disorders | F80-F89 | 5·4 | 3·0 | 2·4 | |
| Behavioral Disorders | F90-F98 | 10·0 | 7·5 | 2·6 | |
| Outpatient Service Use Only (n=41,477) |  |  |  |  | |
| Any Diagnosis |  | 99·9 | 35·9 | 64·1 | |
| Organic Disorders | F00-F09 | 0·2 | <0·1 | 0·1 | |
| Substance use Disorders | F10-F19 | 4·8 | 1·8 | 2·9 | |
| Schizophrenia Spectrum Disorder | F20-F29 | 4·4 | 1·8 | 2·2 | |
| Mood Disorders | F30-F39 | 50·9 | 20·0 | 30·9 | |
| Neurotic Disorders | F40-F48 | 50·7 | 19·1 | 31·6 | |
| Eating Disorder | F50-F59 | 10·1 | 4·5 | 5·5 | |
| Specific Personality Disorder | F60-F69 | 8·0 | 3·7 | 4·3 | |
| Intellectual Disabilities | F70-F79 | 1·7 | 0·5 | 1·2 | |
| Pervasive developmental disorders | F80-F89 | 5·1 | 2·3 | 2·7 | |
| Behavioral Disorders | F90-F98 | 9·2 | 5·9 | 3·3 | |

Note. ^a^: Percentages are relative to the overall number of adult psychiatric service users for each investigation (overall, inpatient and outpatient service use).

***Supplementary analysis 6*** ***Adult psychiatric service use in child (under 13) and adolescent (13 and older) CAMHS users.***

Within this analysis we have stratified CAMHS attendance into those whose first attended CAMHS in childhood (under 13) and those whose first attended was in adolescence (13 and above). Sixty-nine percent of first CAMHS attendance in adolescence. Childhood CAMHS users and Adolescent CAMHS users make up 3·7% (n=13,694) and 8·2% (n=30,614) of the total population. Below we give a breakdown of inpatient admissions and outpatient appointments when stratified by age of first CAMHS attendance; childhood CAMHS users or adolescent CAMHS users.

**Adult Inpatient services.** *Number and total proportion of all inpatient admission.* The total number of adult inpatient admissions was k=45,471. 39·6% (k=18,005) of these adult admissions were from adolescent CAMHS users whereas only 12·6% (k=5,723) were from childhood CAMHS users. Relative to CAMHS non-users, a significantly higher percentage of adolescent CAMHS users had an adult inpatient admission (OR:7·1, CI:6·9-7·4) as did childhood CAMHS users (OR:5·2, CI:4·9-5·5). Relative to childhood CAMHS users, a significantly higher percentage of adolescent CAMHS users had an adult inpatient admission (OR:1·4, CI:1·3-1·5). Both child and adolescent CAMHS users have a vastly higher number of adult inpatient admissions than CAMHS non-users, and adolescent CAMHS users had a higher number of adult inpatient admissions than childhood CAMHS users (See supplementary table 7).

**Supplementary Table 7.** Percentage and odds ratio for adult inpatient admission in CAMHS non-users and CAMHS non-inpatient users relative to CAMHS inpatient users.

| **Number of adult Inpatient admissions** | **CAMHS non-user**  **% (n)** | **Childhood CAMHS Users % (n)** | **Adolescent CAMHS Users % (n)** | **Childhood CAMHS user comparison** | **Adolescent CAMHS user comparison** | |
| --- | --- | --- | --- | --- | --- | --- |
|  |  |  |  | **CAMHS non-Users**  **Risk Ratio**  **(95%CI)** | **CAMHS non-Users**  **Risk Ratio**  **(95%CI)** | **Childhood CAMHS Users Risk Ratio**  **(95%CI)** |
| 0 | 97·5  (321,012) | 88·3  (12,096) | 84·6  (25,905) | Ref | Ref | Ref |
| 1 | 1·3  (4,125) | 4·7  (645) | 6·1  (1,856) | **4**·**1**  (3·8-4·5) | **5**·**6**  (5·3-5·9) | **1**·**3**  (1·2-1·5) |
| 2-5 | 1·0  (3,269) | 5·1  (703) | 6·6  (2,008) | **5**·**7**  (5·3-6·2) | **7**·**6**  (7·2-8·1) | **1**·**3**  (1·2-1·5) |
| 6 + | 0·2  (773) | 1·8  (250) | 2·8  (845) | **8**·**6**  (7·4-9·9) | **13**·**5**  (12·3-14·9) | **1**·**6**  (1·4-1·8) |

*Duration of stay and age of first admission.* Child and Adolescent CAMHS users have a higher number of days spend in inpatient treatment in adulthood (Childhood CAMHS users median:62 days IQR:6-215; Adolescent CAMHS users median:78 days IQR:11-240; CAMHS non-users median:48 days IQR:5-163; X^2^ = 101·3, p < 0·001). Only adolescent CAMHS users have a higher number of days per admission (Childhood CAMHS users median:32 days IQR:4.5-79; Adolescent CAMHS users median:37·7 days IQR:8-86·6; CAMHS non-users median:32 days IQR:4·5-81; X^2^ = 53·4, p < 0·001). Adolescent CAMHS users have their first adult inpatient admission roughly 7 months year earlier than Childhood CAMHS users and two years earlier than CAMHS non-users (Childhood CAMHS users’ median age:21·1 IQR:19·2-23·5; Adolescent CAMHS users median age:20·5 IQR:18·9-22·9; and CAMHS non-users median age:22·4 IQR:20·2-24·6, X^2^ = 480·4, p < 0·001).

**Outpatient service use.**  *Number and total proportion of all outpatient appointments attended.* The total number of outpatient appointments attended was k=1,345,060. 42·3% (k=568,892) of these adult outpatient appointments were from adolescent CAMHS users and 10·8% (k= 145,347) were from childhood CAMHS users. Relative to 10·3% of CAMHS non-users, a significantly higher percentage of both adolescent CAMHS users (51·9%, OR:9·4, CI:9·2-9·7) and childhood CAMHS users (36·0%, OR:4·9, CI:4·7-5·1) had an adult outpatient appointment. Relative to childhood CAMHS users, a significantly higher percentage of adolescent CAMHS users had an adult outpatient appointment (OR:1·9, CI:1·8-2·0). Both child and adolescent CAMHS users have a vastly higher number of adult outpatient appointment than CAMHS non-users, and adolescent CAMHS users had a higher number of adult outpatient appointment than childhood CAMHS users (See supplementary table 8).

**Supplementary Table 8.** Percentage and odds ratio for the number of adult outpatient appointments attended in CAMHS non-users and CAMHS non-inpatient users relative to CAMHS inpatient users.

| **Number of adult Inpatient admissions** | **CAMHS non-user**  **% (n)** | **Childhood CAMHS Users % (n)** | **Adolescent CAMHS Users % (n)** | **Childhood CAMHS user comparison** | **Adolescent CAMHS user comparison** | |
| --- | --- | --- | --- | --- | --- | --- |
|  |  |  |  | **CAMHS non-Users**  **Risk Ratio**  **(95%CI)** | **CAMHS non-Users**  **Risk Ratio**  **(95%CI)** | **Childhood CAMHS Users Risk Ratio**  **(95%CI)** |
| **0** | 89·7  (295,292) | 64·0  (8,758) | 48·1  (14,716) | Ref | Ref | Ref |
| **1** | 2·9  (9,498) | 6·7  (913) | 7·5  (2,306) | **3**·**2**  (3·0-3·5) | **4**·**9**  (4·6-5·1) | **1**·**5**  (1·4-1·6) |
| **2-5** | 2·7  (8,758) | 8·4  (1,145) | 10·8  (3,310) | **4**·**4**  (4·1-4·7) | **7**·**6**  (7·3-7·9) | **1**·**7**  (1·6-1·8) |
| **6-10** | 1·2  (4,066) | 5·1  (698) | 6·7  (2,053) | **5**·**8**  (5·3-6·3) | **10**·**1**  (9·6-10·7) | **1**·**8**  (1·6-1·9) |
| **11-25** | 1·6  (5,174) | 5·9  (814) | 9·7  (2,971) | **5**·**3**  (4·9-5·7) | **11**·**5**  (11·0-12·1) | **2**·**2**  (2·0-2·4) |
| **26-50** | 1·0  (3,139) | 4·1  (567) | 6·8  (2,080) | **6**·**1**  (5·6-6·7) | **13**·**3**  (12·5-14·1) | **2**·**2**  (2·0-2·4) |
| **51-100** | 0·6  (1,997) | 3·2  (443) | 5·5  (1,685) | **7**·**5**  (6·7-8·3) | **16**·**9**  (15·8-18·1) | **2**·**3**  (2·0-2·5) |
| **101+** | 0·4  (1,255) | 2·6  (356) | 4·9  (1,493) | **9**·**6**  (8·5-10·8) | **23**·**9**  (22·1-25·8) | **2**·**5**  (2·2-2·8) |
